# A novel real-world data methodology for lymphoma outcome classification: the real-world Lugano study

**DOI:** 10.57264/cer-2025-0134

**Published:** 2026-03-12

**Authors:** Richard Scott Swain, Andrew Klink, Parisa Asgarisabet, Kristin M Zimmerman Savill, Bindu Kalesan, Alexandrina Balanean, Harlen Hays, Jill Kaufman, Lindsay McAllister, Courtney Omary, Hsing-Ting Yu, JaLyna Laney, Nicholas C Richardson, Catherine C Lerro, Fatima Rizvi, Jonathon Vallejo, Kun Wang, Marc R Theoret, Donna R Rivera, Bruce A Feinberg

**Affiliations:** 1 Cardinal Health, Dublin, OH 43017, USA; 2 Office of Oncologic Disease, US Food & Drug Administration, Silver Spring, MD 20993, USA; 3 Oncology Center of Excellence, US Food & Drug Administration, Silver Spring, MD 20993, USA; 4 Office of Biostatistics, US Food & Drug Administration, Silver Spring, MD 20993, USA

**Keywords:** BICR, blinded independent central review, clinical trial alignment, concordance analysis, DLBCL, diffuse large B-cell lymphoma, Real-world data, real-world evidence, real-world Lugano, RWE, RWD, rwLugano, treatment response classification

## Abstract

**Aim:**

In oncology trials, blinded independent central review (BICR) is the standard for treatment response classification. Real-world data methodologies that align with BICR may reduce misclassification in real-world evidence (RWE) studies and enhance reproducibility, increasing value of RWE. We aimed to develop and validate a novel real-world data-based methodology – real-world Lugano (rwLugano) – for assessing lymphoma response to align with clinical trials.

**Materials & methods:**

We conducted a retrospective, multisite chart abstraction study using Cardinal Health Practice Research Network (PRN) sites to identify adults with diffuse large B-cell lymphoma initiating first-line (1L) therapy from 1 January 2015, through 31 December 2022, in US community oncology. Sites collected patient characteristics and PET/CT scans at baseline and first response. Two radiologists independently classified responses; a medical oncologist adjudicated discordances.

**Results:**

We compared initial treatment responses using three methods: physician-charted from electronic health records, rwLugano-derived per Lugano 2014 and BICR-adjudicated per Lugano 2014. Agreement was assessed via percentage concordance, kappa (κ), and multivariable generalized linear mixed modeling for assigning complete response (CR). Among 178 patients, CR rates were 63.5% (physician-charted), 81.5% (rwLugano) and 83.1% (BICR). Compared with BICR, rwLugano showed higher agreement (87.9%, κ = 0.52) than physician-charted (77.0%, κ = 0.40). The generalized linear mixed modeling analyses identified clinical factors associated with concordance: for physician charted assessments, greater numbers of extranodal sites increased agreement with BICR (OR 1.92), while MYC mutation (OR 0.38) and anemia (OR 0.37) reduced agreement. For rwLugano, nonprivate insurance was associated with higher agreement (odds ratio [OR]: 4.40), whereas MYC mutation reduced agreement (OR: 0.26).

**Conclusion:**

rwLugano improves real-world lymphoma response classification, aligning with BICR and supporting more accurate, reproducible RWE for clinical and regulatory decision-making. Using methods BICR and rwLugano may provide opportunities to minimize outcome misclassification and improve comparability of clinical trial and clinical practice approaches.

## Plain language summary

**What is this article about?:**

In real-world lymphoma research, can a novel treatment response classification be leveraged to closely emulate assessment methods used in a clinical trial?

**What were the results?:**

Compared with blinded independent central review (BICR)-adjudicated complete response (CR) in patients with aggressive lymphoma, physician-charted assessment underestimated CR, whereas classification via the novel real-world Lugano (rwLugano) method was similar to BICR. Initial CR was lower for physician-charted (63.5%) versus rwLugano-derived (81.5%) and BICR-adjudicated (83.1%) methods – the latter 2 may minimize end point misclassification and improve comparability with clinical trials.

**What do the results mean?:**

The novel rwLugano approach has potential to classify treatment outcomes consistent with those resulting from clinical trial approaches.

## Background & rationale

The 21st Century Cures Act, designed to accelerate medical development to bring innovations to patients faster and more efficiently, required the US FDA to develop a framework for real-world evidence (RWE) in regulatory decision-making [1,2]. The use of RWE to complement clinical trial data is increasing in oncology. However, challenges in the availability of real-world oncology end points comparable with clinical trial end points can impact the utility of RWE in supporting clinical and regulatory decision-making.

In oncology, drug approvals can be based on intermediate clinical end points of objective response rate or complete response (CR) rate along with time-to-event clinical end points such as progression-free survival and overall survival (OS) [3,4]. In clinical trials for patients with diffuse large B-cell lymphoma (DLBCL), treatment response is measured using standardized criteria that incorporates clinical, radiologic and diagnostic information [5]. The currently used criteria, Lugano 2014, are primarily based on positron emission tomography (PET) and computed tomography (CT) imaging at baseline, during, and after treatment to evaluate metabolic activity and tumor size.

A subtype of non-Hodgkin lymphoma, DLBCL is fluorodeoxyglucose (FDG)-avid. In 2009 in Deauville, France, a 5-point-scale (5-PS) for FDG uptake was recommended as the standard response assessment reporting tool at the First International Workshop on PET in Lymphoma [6]. In 2011, the International Conference on Malignant Lymphoma (ICML) met in Lugano, Switzerland, to update recommendations for evaluating, staging and assessing lymphoma treatment response using FDG-based PET/CT [5]. In addition to lesion size, the resulting Lugano 2014 criteria employ the Deauville 5-PS scoring system to quantify the standardized uptake value (SUV) of the PET nuclear isotope radio tracer in disease and background tissue sites to determine the extent of disease and response to therapy (Appendix Table 1) [5]. The 5-PS scoring system is underreported in community oncology practice [7].

The evaluation of treatment efficacy per the Lugano 2014 criteria defines response categorization as 1 of 4 possible outcomes: complete metabolic response (CR), partial metabolic response (PR), stable disease (SD) or progressive disease (PD) [5]. In oncology clinical trials with a primary response-based end point, assignment of response through independent interpretation of PET/CT imaging via blinded independent central review (BICR) is often leveraged, with the goal of increasing objectivity and reducing potential bias [8].

In routine clinical practice, disease management, response interpretation and Lugano criteria reporting are highly variable, and BICR is generally not used. Real-world clinical response determination may account for information from digital imaging reviews, narrative scan reports and clinical and laboratory findings. The RWD-based response end points may be confounded by issues such as misclassification and irreproducibility. Hence, direct comparisons of real-world and clinical trial outcome assessments are challenging [5,9,10]. The development and validation of a clinically relevant measure of lymphoma treatment response that approximates clinical trial methods may increase the utility of RWE in support of evaluating new therapies or therapeutic indications for patients with lymphoma. Additionally, such a measure could represent a new benchmark for lymphoma response assessment outside of clinical trials. Toward this aim, we created a novel methodology for lymphoma response assessment termed real-world Lugano (rwLugano).

## Research objective & outcome measures

Our objective was to develop a method of evaluating treatment response in patients with DLBCL who initiated first-line (1L) treatment with chemoimmunotherapy using RWD. Using our novel rwLugano algorithm, which applies the Lugano 2014 classification using the Deauville 5-PS, we compared physician-charted and rwLugano-derived response assessments and then compared both to BICR-adjudicated assessments via percentage agreement, Cohen’s kappa (κ) concordance and odds ratios (ORs) with 95% CIs. These were calculated using multivariable generalized linear mixed modeling (GLMM) for classification of CR (primary end point), PR, no response (NR)/SD and PD.

## Materials & methods

### Study design, setting & population

We conducted a retrospective, multisite observational study using the Cardinal Health Practice Research Network (PRN) to compare methods for assessing response to therapy in DLBCL. By focusing on initial response to 1L systemic therapy, we prioritized evaluating the feasibility and validity of a new response assessment methodology over clinical trial end points like progression-free survival and OS. Patients were identified at six sites, with eligibility limited to adults diagnosed with histologically confirmed DLBCL who initiated 1L therapy between 1 January 2015 and 31 December 2022. The sites identified the first eligible patient and abstracted additional patients chronologically until the end of the period or until the 40-patient per-site limit was reached.

Comparisons were conducted among three methods of response classification: physician-charted, abstracted from electronic health records (EHRs), rwLugano-derived, per Lugano 2014 criteria using PET/CT scans and reports and BICR-adjudicated. For BICR, two independent radiologists reviewed outcome classifications, and a medical oncologist adjudicated discordances in response assignment. At least 6 months of postdiagnostic follow-up and two PET/CT scans, including a baseline scan within 8 weeks prior to initiating 1L therapy and initial response assessment scan within 24 weeks, were required. Patients were excluded if other malignancies or central nervous system metastases were present, or if they had enrolled in a clinical trial during 1L treatment of DLBCL. Full inclusion and exclusion criteria are displayed in Appendix Table 2.

### Data source

The Cardinal Health PRN is research-focused. As of January 2025, it comprised 56 community-based oncology practices with 378 participating physicians [11]. Practices are contracted per study, and research staff complete the prospectively designed electronic case report forms (eCRFs) – unique to each study – for their patients, enabling comprehensive EHR data collection by familiar clinicians. The PRN data collection capabilities include radiology scans, pathology reports, billing data and patient-reported outcomes. This allows provider, treatment and clinical data to be captured from archived records, mirroring clinical trial procedures with strict selection criteria and standardized data collection.

### Data collection & outcome definitions

Research staff entered data related to patient demographic and clinical characteristics, 1L therapy, clinical outcomes and scan/radiology report details into the study eCRFs using the Fountayn Electronic Data Capture system. Additionally, deidentified radiologic images from a minimum of two scans (one baseline and one follow-up) per patient were provided for BICR response assessment.

### Physician-charted response assessment

Physician-charted initial response to 1L therapy was abstracted as CR, PR, SD/NR or PD based on the medical record.

### RwLugano

**Figure 1.**
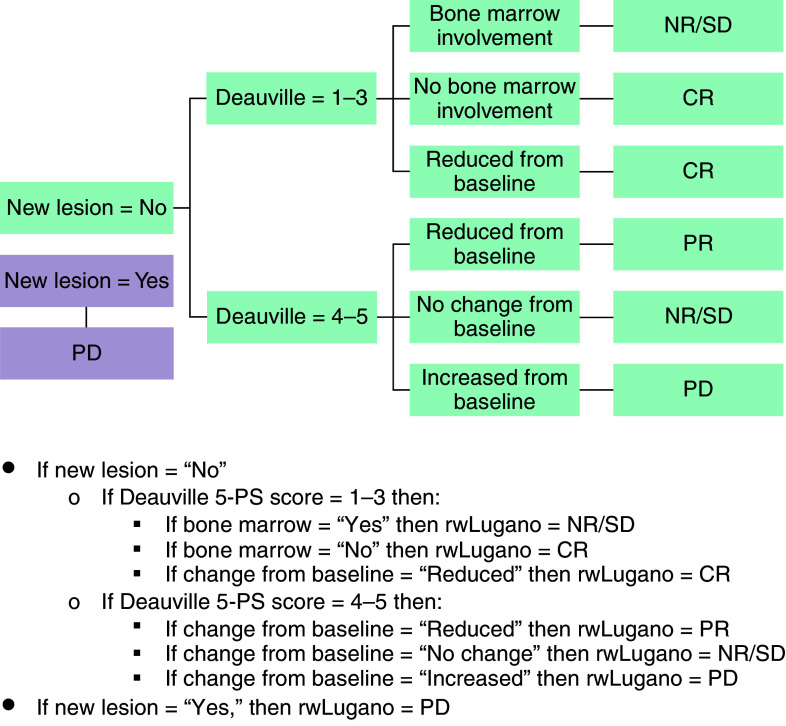
rwLugano algorithm. 5PS: 5-point scale; CR: Complete response; NR/SD: No response/stable disease; PD: Progressive disease; PR: Partial response; rw: Real-world.

Data elements pertaining to the Lugano 2014 criteria were abstracted. Next, an algorithm was developed, from which we derived the rwLugano method (Appendix Table 1 & Figure 1) [10]. Additionally, we devised the rwDeauville methodology to impute missing 5-PS scores into the rwLugano algorithm. The 5-PS score was considered complete if it was recorded in the imaging report or if the SUVs for mediastinum, liver, and primary tumor sites were recorded (SUV can be used to calculate 5-PS). When neither was the case, we calculated rwDeauville scores by replacing the missing SUVs with those consistent with high-normal levels for background (SUV = 1), mediastinum (SUV = 3) and liver (SUV = 5) sites [12]. For further validation, the primary tumor 5-PS score was also calculated using rwDeauville and compared with baseline PET/CT values. The rwDeauville methodology is shown in Appendix Figure 1.

### Blinded independent central review (BICR)

Two independent radiologists compared baseline and initial response scans obtained at 8–24 weeks after 1L therapy initiation without additional clinical information, and any discordant classifications were assessed by an adjudicator (medical oncologist) for consensus.

### Study conduct & ethics

This study (protocol 1340909) followed the Strengthening the Reporting of Observational Studies in Epidemiology (STROBE) guidelines [13] and was approved with a waiver of consent by the Western Institutional Review Board (WIRB)-Copernicus Group (WCG).

### Statistical analysis

Assuming nonproportional response, 4 × 4 comparison (response categories CR, PR, NR/SD and PD per two methods), 2-sided α = 0.05, and 90% power, we estimated requiring a minimum of 164 patients for distinguishing between concordance of κ1 = 0.60; 95% CI: 0.53–0.68 (estimated κ value with concordance between rwLugano-derived and physician-charted response based upon prior studies) and κ2 = 0.80; 95% CI: 0.73–0.85 (minimum κ value with concordance between rwLugano-derived and BICR-adjudicated response to show stronger agreement). The target minimum was 180 patients, allowing for at least 360 scans or 180 scan pairs and accounting for 10% attrition due to missing data or data quality issues.

Patient demographic, clinical, and treatment characteristics were summarized descriptively. By using overall percentage agreement and κ, the primary objective assessed concordance between physician-charted and BICR-adjudicated responses and between rwLugano-derived and BICR-adjudicated responses. A generalized linear mixed model (GLMM) estimated whether physician-charted and rwLugano-derived methods (CR vs non-CR) were classifying responses as statistically significantly different from BICR-adjudicated responses, and logistic regression examined potential influences of baseline patient characteristics on the accuracy of classification.

We analyzed the impact of covariates and raters on treatment response prediction using GLMM. The initial model used a probit link for binary outcomes and treated patients and raters as random effects. Subsequent models explored both additional random effects and conditional likelihood. Core random effects for each scan were the patient and rater, with the PRN site as the potential third. Conditional likelihood included significant variables based on descriptive analysis and expected patient age, time between scans and tumor cell of origin. Additional clinical and demographic information was considered based on descriptive statistics in the primary and secondary analyses.

## Results

There were 178 eligible patients with DLBCL included from six PRN practices comprising 77 physicians. The practices were located throughout the US; there were one each from the Midwest, Northeast and West, and three from the South US regions (Table 1). Among the patients, the mean age at 1L therapy initiation was 66.5 years and most were male (59.0%), with race reported as White (77.0%), Black (10.1%), unknown (10.1%) or Asian (2.8%); 7.3% had Hispanic ethnicity. Additionally, 172 patients (96.6%) had Eastern Cooperative Oncology Group performance status (ECOG PS) scores available, 91.3% of which were 0 to 1. Among 154 patients (86.5%) with known Ann Arbor stage at 1L initiation, 57.1% had stage III–IV. Among all 178 patients, 19.7% had bulky disease and 95.5% received 1L rituximab, cyclophosphamide, doxorubicin, vincristine and prednisone (R-CHOP). The median (25th–75th percentile [P25–P75]) time from 1L initiation to initial PET/CT scan was 14.1 (10.6–19.0) weeks, and median (P25–P75) follow-up from 1L initiation was 25.6 (16.8–43.8) months. The PRN site characteristics are presented in Appendix Table 3.

**Table 1.** Patient baseline demographic, clinical, and treatment characteristics.

Demographic characteristics (total eligible patients = 185)	
Patients with reviewable PET/CT scans/reports	N = 178
Study site location, n (%)	
South (AL, AR, DC, FL, GA, KY, LA, MS, NC, OK, SC, TN, TX, VA, WV)	3.0 (50.0)
Midwest (IA, IL, IN, KS, MI, MN, MO, ND, NE, OH, SD, WI)	1.0 (16.7)
Northeast (CT, DE, MA, MD, ME, NH, NJ, NY, PA, RI, VT)	1.0 (16.7)
West (AK, AZ, CA, CO, HI, ID, MT, NM, NV, OR, UT, WA, WY)	1.0 (16.7)
Age at DLBCL diagnosis, years	
Mean (SD)	66.4 (12.8)
<65	71.0 (39.9)
65–74	55.0 (30.9)
75+	52.0 (29.2)
Sex, n (%)	
Male	105.0 (59.0)
Female	73 (41.0)
Race, n (%)	
American–Indian or Alaska Native	0.0 (0.0)
Asian	5.0 (2.8)
Black or African–American	18.0 (10.1)
Native Hawaiian or Other Pacific Islander	0.0 (0.0)
White	137.0 (77.0)
Unknown	18.0 (10.1)
Ethnicity, n (%)	
Hispanic or Latino	13.0 (7.3)
Not Hispanic or Latino	143.0 (80.3)
Unknown	22.0 (12.4)
Insurance, n (%)	
Medicare	102.0 (57.3)
Medicaid	5.0 (2.8)
Commercial	59.0 (33.2)
Military	5.0 (2.8)
Self-pay	2.0 (1.1)
Unknown	5.0 (2.8)
**Patient status at data collection, n (%)**	
Alive	**165.0 (92.7)**
In remission	139.0 (84.2)
Relapsed and currently receiving active therapy	8.0 (4.9)
Receiving only palliative care	2.0 (1.2)
Referred to hospice	2.0 (1.2)
Unknown, lost to follow-up	7.0 (4.2)
Other	7.0 (4.2)
Deceased	**13.0 (7.3)**
Disease progression	3.0 (23.1)
Unknown, data not available	8.0 (61.5)
Other	2.0 (15.4)
**Clinical characteristics**	
Duration of follow-up in months, mean (SD)	31.3 (19.4)
Cell of origin identified in report, n (%)	
Germinal center B-cell like	75.0 (42.1)
Non-germinal center B-cell like	55.0 (30.9)
Activated B-cell like	11 (6.2)
Unclassified	13.0 (7.3)
None	24.0 (13.5)
**ECOG PS score at 1L therapy initiation, n (%)**	
Numeric	
0	117.0 (65.7)
1	40.0 (22.5)
2	14.0 (7.9)
3	1.0 (0.6)
Missing	6.0 (3.4)
Categoric	
0/1	157.0 (88.2)
2+	15.0 (8.4)
Missing	6.0 (3.4)
Ann Arbor stage at 1L therapy initiation, n (%)	
Stage I	24.0 (13.5)
Stage II	42.0 (23.6)
Stage III	38.0 (21.3)
Stage IV	50.0 (28.1)
Missing	24.0 (13.5)
Cytopenia at 1L therapy initiation, n (%)^†^	
Neutropenia	3.0 (1.7)
Anemia	49.0 (27.5)
None of the above	128.0 (71.9)
Disease characteristics at 1L therapy initiation^†^	
Extranodal disease, n (%)	64.0 (36.0)
Extranodal sites, n, mean (SD)	*1.5 (0.9)*
Bulky disease (≥7 cm), n (%)	35.0 (19.7)
Bone marrow involvement, n (%)	14.0 (7.9)
None of the above, n (%)	82.0 (46.1)
Comorbidities prior to 1L therapy initiation, n (%)^†^	
Anemia	49.0 (27.5)
Diabetes (with and without complications)	36.0 (20.2)
Hypertension	94.0 (52.8)
Dyslipidemia/hypercholesterolemia	54.0 (30.3)
Heart disease (cardiovascular disease, congestive heart failure, coronary artery disease, myocardial infarction)	38.0 (21.3)
Mental illness (anxiety, depression, dementia)	30.0 (16.9)
Joint disorders (arthritis, gout)	32.0 (18.0)
**Treatment characteristics**	
1L therapy for DLBCL, n (%)	
R-CHOP	170.0 (95.5)
Other	8.0 (4.5)
Time from baseline scan to 1L therapy initiation, weeks	
Mean (SD)	2.2 (1.8)
Median, P25–P75	1.7, 0.9–3.0
Min, max	0.1, 8.0
Time from 1L therapy initiation to initial response PET/CT scan, weeks	
Mean (SD)	15.0 (4.9)
Median, P25–P75	14.1, 10.9–19.0
Min, max	8.0, 23.7

1L: First-line; CT: Computed tomography; DLBCL: Diffuse large B-cell lymphoma; ECOG PS: Eastern Cooperative Oncology Group performance score; max: Maximum; min: Minimum; P25–P75: 25th through 75th percentiles; PET: Positron emission tomography; R-CHOP: Rituximab, cyclophosphamide, doxorubicin, vincristine, prednisone; SD: Standard deviation.

† Not mutually exclusive.

**Figure 2.**
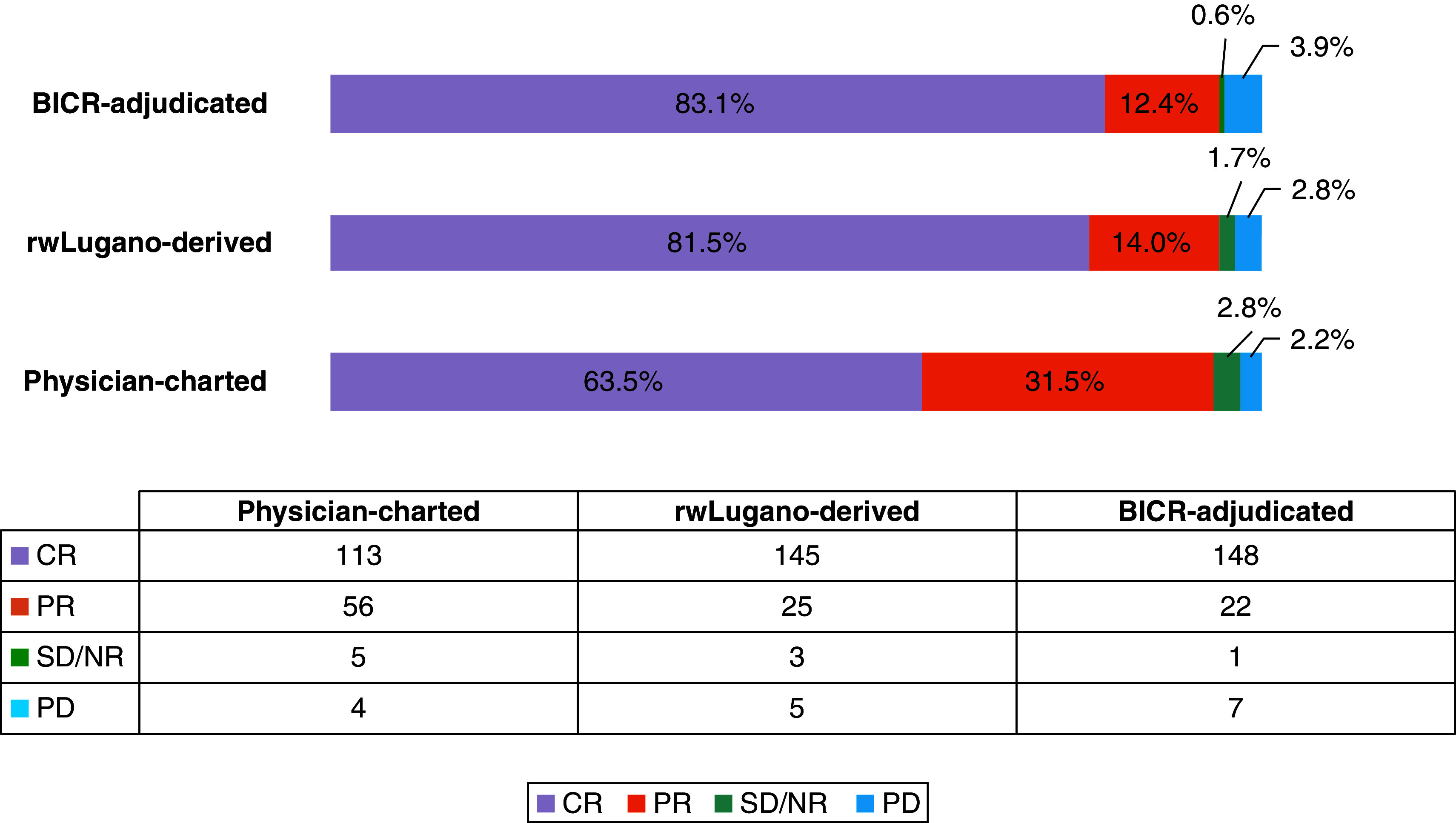
Assignment of complete response at initial response assessment for each classification method (n = 178). BICR: Blinded independent central review; CR: Complete response; NR/SD: No response/stable disease; PD: Progressive disease; PR: Partial response; rw: Real-world.

Assessment of initial response via BICR classified 148 patients as having CR (83.1%), 22 as PR (12.4%), 1 as NR/SD (0.6%) and 7 as PD (3.9%). Assignment of CR was proportionately lower for physician-charted (63.5%) compared with rwLugano-derived (81.5%) and BICR-adjudicated (83.1%) methods (Figure 2). Table 2 shows that compared with BICR-adjudication, physician-charted assessment underestimated CR (agreement: 77.0%; κ = 0.40; OR: 0.23, 95% CI: 0.12–0.43) whereas rwLugano-derived assessment (agreement: 87.9%; κ = 0.52; OR: 1.19, 95% CI: 0.61–2.33) performed similarly to BICR. The 5-PS score was missing in 30 initial response PET/CT reports (16.9%). Among 148 scans with complete 5-PS data (83.2%), rwDeauville scores were concordant with 5-PS scores for 127 (87.6%, κ = 0.82, 95% CI: 0.74–0.90) (Appendix Table 4). Further, among patients with complete 5-PS scores, using rwDeauville in place of 5-PS changed response classification for nine scans (6.0%), also changing objective response rate for two scans (1.4%). Based on these results, rwDeauville was used to impute missing 5-PS scores. Lastly, adjudication of discordant BICR assessments was required for 17.4% of responses between the two radiologists (Table 3), with discordances most frequently related to interpretations of bone marrow activity and/or new sites of disease. The overall percentage agreement was 84.3%.

**Table 2.** Multivariable analysis of association between physician-charted and rwLugano-derived response versus blinded independent central review with treatment response (n = 178).

Association between independent variables and CR: multivariable model (yes/no)^†^	
Reviewer (main independent variable)	OR (95% CI)
BICR-adjudicated (n 178)^‡^	Reference
Physician-charted (n = 178) (agreement: 77.0%, κ = 0.40)^‡^	0.23 (0.12, 0.43)
rwLugano-derived (n = 178) (agreement: 87.9%, κ = 0.52)^‡^	1.19 (0.61, 2.34)
**Provider ID**	
02 (n = 37)	Reference
03 (n = 31)	2.31 (0.63, 8.40)
04 (n = 39)	2.31 (0.76, 7.04)
06 (n = 20)	1.68 (0.44, 6.49)
07 (n = 11)	0.12 (0.03, 0.52)
08 (n = 40)	2.35 (0.77, 7.19)
**Stage at diagnosis**	
Stage I (n = 24)	Reference
Stage II (n = 42)	0.16 (0.03, 0.71)
Stage III (n = 38)	0.33 (0.07, 1.57)
Stage IV (n = 50)	0.16 (0.04, 0.67)
Not available (n = 24)	0.08 (0.02, 0.40)
**Comorbidities**	
Anemia	
No (n = 129)	Reference
Yes (n = 49)	0.39 (0.17, 0.90)
Heart disease (cardiovascular disease, congestive heart failure, coronary artery disease, myocardial infarction)	
No (n = 140)	Reference
Yes (n = 38)	0.25 (0.11, 0.59)

† We used GLMM analysis to determine the further impact of covariates and raters upon the level of agreement regarding predictors of treatment response. The model used a probit link for the binary categorical outcomes of agreement vs nonagreement for each scan. The initial model did not consider any additional covariates and treated both the patient and the raters as random effects. Subsequent models explored both additional random effects and conditional likelihood. Core random effects in the model were the patient as well the rater for each scan, with the site as a potential additional random effect. Conditional likelihood in the model included clinically and statistically significant variables based on the descriptive analysis and were expected to include age of the patient at study initiation, time between imaging scans, and tumor cell of origin. Additional clinical and demographic information was considered based on descriptive statistics in the primary and secondary analyses.

‡ κ1 is the difference between rwLugano-derived and physician-charted values and κ2 is the difference between rwLugano-derived and BICR-adjudicated values.

**Treatment response end points:**

Physician-charted response: 1L therapy response as charted in the medical record; the physician-charted response with the date closest to the scan date for rwLugano-derived and BICR-adjudicated assessments was used for pairwise comparison.

rwLugano-derived response: Calculated response based on Lugano classification components available on pretreatment (baseline) scans compared with scans on-treatment, with at least one on-treatment scan performed at initial response within 12 weeks; additional 1L on-treatment time points were evaluated as available for up to 6 months. The Lugano classification uses the terms complete metabolic response and partial metabolic response synonymously with CR and PR. In this study, CR and PR were used. The Lugano 5-PS is based on the SUV of the most metabolically active lesion: no uptake above background; uptake ≤ mediastinum; uptake > mediastinum but ≤ liver; uptake moderately increased > liver; markedly increased uptake above liver at any site and/or new lesions.

BICR-adjudicated response: Assigned by two independent radiologists comparing pretreatment (baseline) scans with on-treatment scans, with at least 1 on-treatment (1L) scan performed at initial response within 12 weeks, using Lugano criteria; no additional clinical information was included in this assessment. Additional 1L on-treatment timepoints were evaluated as available for a given patient up to 6 months. Where there were discordant response classifications between the two independent radiologists, cases were reevaluated by both radiologists and discussed together to agree on 1 response classification for the on-treatment scan in question.

1L: First-line; 5-PS: 5-point scale; BICR: Blinded independent central review; CR: Complete response; GLMM: Generalized linear mixed model; ID: Identification; κ: Cohen’s kappa; NR/SD: No response/stable disease; OR: Odd ratio; PD: Progressive disease; PR: Partial response; rw: Real-world; SUV: Standardized uptake value.

**Table 3.** Blinded independent central review concordance between radiologists.

	Radiologist 2				
Radiologist 1	CR	PR	NR/SD	PD	IND
CR	122	3	1	1	9
PR	6	20	1	1	1
NR/SD	1	1	2	0	0
PD	2	1	0	3	0
IND	0	0	0	0	3
Overall percentage agreement (total concordance/total sample)	84.3%				
Percentage agreement considering indeterminate as disagreement κ (95% CI)	82.6% 0.66 (0.53–0.78)				

BICR: Blinded independent central review; CR: Complete response; IND: Indeterminate; κ: Cohen’s kappa; NR/SD: No response/stable disease; PD: Progressive disease; PR: Partial response.

Multivariable logistic regression (Appendix Table 5) showed that the number of extranodal disease sites increased the odds of agreement between physician-charted classification and BICR adjudication (OR: 1.92, 95% CI: 1.1–3.4), whereas *MYC* mutation (OR: 0.38, 95% CI: 0.15–0.98) and anemia (OR: 0.37, 95% CI: 0.17–0.82) reduced the likelihood of concordance. Between rwLugano-derived and BICR adjudication, nonprivate health insurance (OR: 4.40, 95% CI: 1.4–13.7) and *MYC* mutation (OR: 0.26, 95% CI: 0.09–0.76) were associated with agreement. The full univariate and multivariable analysis results are in Appendix Table 6. A subgroup analysis based on initial BICR concordance found that both physician-charted (overall agreement: 73.5%, κ = 0.44 when concordant and 61.3%, κ = 0.39 when discordant) and rwLugano-derived (overall agreement: 85.7%, κ = 0.50 when concordant and 74.2%, κ = not evaluable [NE] when discordant) responses were numerically more accurate when BICR reviewers were initially concordant. Additionally, rwLugano was numerically more accurate (Appendix Tables 7–9).

## Discussion

In this observational study, we found that using our novel rwLugano method resulted in CR classification that was more closely correlated with the clinical trial response assessment method of BICR than with physician-charted assessment. Our analysis demonstrated that physician-charted assessment of patients with DLBCL tend to underestimate CR rates for 1L initial response compared with BICR-adjudicated assessment. Moreover, BICR is feasible and can be performed with RWD as a further refinement to align RWE outcomes with those of clinical trials. Various sensitivity analyses were also conducted to identify factors impacting rwLugano classification. For example, rwLugano scores were compared for patients who had complete 5-PS scores available versus those for whom rwDeauville scores were calculated. Among the patients with complete 5-PS scores, we had hypothesized a stronger correlation between rwLugano and BICR response assessment than we observed. Weighted κ is differentially influenced by larger discordance (i.e., CR vs PD discordance lowers κ more than does CR vs PR discordance), and rwLugano-derived scores had more discordant outliers (i.e., BICR-adjudicated CR and rwLugano-derived PD) with BICR than physician-charted responses. Excluding such outliers would have had little impact on concordance estimates between physician-charted and BICR-adjudicated assessments, whereas calculation of rwLugano would have been more affected. Notably, the substantial missingness of the 5PS score – absent in 17% of routine practice PET/CT reports – reinforces that physician charted assessments alone are insufficient for reliable response classification. Therefore, the development and implementation of rwDeauville were essential, enabling us to recover a core Lugano component that is routinely underreported in community oncology. Importantly, the rwLugano methodology, supported by rwDeauville, maintained strong alignment with BICR despite this missingness, demonstrating that algorithmic correction can mitigate RWD limitations and strengthen the validity of real-world response end points.

Additionally, GLMM results were less affected by outliers, revealing that physician-charted response underestimated CR versus BICR adjudication; rwLugano differed from physician-charted responses, but not significantly from BICR-adjudicated responses. Compared with BICR, physician-charted and rwLugano-derived assessments differed.

When interpreting these findings, it is important to contextualize the κ statistics within real-world oncology research. Retrospective RWD studies inherently face constraints such as variable documentation quality, heterogeneous PET/CT reporting and missing 5PS elements, all of which limit the achievable concordance among methods. In this setting, even moderate κ values represent meaningful improvement, particularly relative to the known variability of physician charted assessments. Thus, the value of the rwLugano methodology lies less in attaining absolute agreement – which is not realistic for retrospective –imaging based RWE – and more in its improved alignment with BICR. This strengthens reproducibility and enhances the comparability of real-world response assessments with clinical trial standards.

We have demonstrated that the RWD-based response assessment methods for lymphoma, rwLugano and BICR may be used to design observational RWE studies. Our findings may complement clinical trial data used for regulatory, clinical and patient decision-making, and wider adoption of similarly robust methods may increase RWE generalizability and reproducibility. Questions foundational to this research concerned whether our data source was fit-for-purpose and whether data abstraction was feasible, and study findings supported use of these data to corroborate our methodologic hypothesis. We found that after digital deidentification, data and radiologic images from community practices could be utilized for rwLugano assessment and BICR adjudication. However, we did not anticipate the substantial amount of missing data for the rwLugano classification – specifically the 5-PS scores – which required creating and validating an intermediate methodology, i.e., rwDeauville. Additionally, due to technologic barriers, obtaining digital PET/CT scans was challenging despite their presence in the EHRs. Our process involved activating PRN site staff, radiologists and the research team through identifying, contracting, training and coordinating as appropriate – much like clinical trial site activation. Nevertheless, we obtained baseline and follow-up PET/CT scans for 96.2% of the patients.

Although we intended to standardize DLBCL response assessment with our novel rwLugano algorithm, we now think that clinical (nonmethodologic) factors may explain why physicians underestimated CR at initial assessment. For example, some clinicians wait for confirmation from a subsequent scan before assigning CR. Additionally, the number of extranodal sites was associated with increased accuracy of physician-charted responses, whereas anemia and *MYC* mutation were associated with decreased accuracy. The *MYC* mutation and anemia can affect FDG uptake on PET/CT, making it difficult for nonradiologists to interpret, and extranodal sites are more familiar to oncologists, allowing for easier assessment.

Our evaluation of rwLugano builds upon similar methodologic studies in solid tumors [14–16]. For example, we have previously described our development of rwRECIST, a novel methodology for solid tumor response assessment that applies RECIST v1.1 guidelines based on lesion measurements from PET/CT scans/reports [17]. Across three solid tumor indications, rwRECIST assessment aligned better with published clinical trials than did physician assessments [17]. Both RWD-based methodologies, rwLugano and rwRECIST, warrant further evaluation to quantify their utility and potential sources of bias across various clinical settings. Nevertheless, establishment of RWD assessment methods may reduce variability and bias, thereby standardizing constructs in oncology and hematology research and practice.

### Limitations

This study's retrospective nature – with data collection from EHRs and PET/CT scans rather than a prospective, controlled process – may have introduced selection bias. Additionally, data accuracy and completeness relied on the quality of the source documentation; consequently, inconsistent or incomplete records may have led to response misclassification. Further, the 178-patient sample may not have fully represented the broader patient population, potentially affecting generalizability. Despite the reviews by two independent radiologists and an adjudicator, interrater variability in CR classification may have impacted the agreement rates and κ concordance. Moreover, rwLugano concordance with BICR may have been overstated because both assessments relied on the same PET/CT scans, long term outcomes were not evaluated and replacing missing SUV data through imputation could have introduced bias.

Finally, the timing of response assessments is highly variable in routine care, and not prescriptive as it is in clinical trials, introducing another factor that would require redress if the described methods were to be used for regulatory submissions. Future efforts might evaluate the validation of rwLugano outside of US community oncology practices (e.g., academic centers or international locations), improving its utility.

## Conclusion

Clinical practice-supplied RWD can bridge evidence gaps between clinical trials and practice; specifically, patients treated in real-world settings may differ from those in clinical trials – who may be older, sicker, more diverse, less treatment-adherent and have less access to care [18]. Moreover, treatment effectiveness in clinical practice may be lower than its efficacy under the ideal conditions of a clinical trial. Accordingly, results from studies incorporating RWD can enhance the generalizability of clinical trial findings.

Using RWD, we developed an algorithm to capture treatment response per Lugano 2014 criteria, which may better align with BICR adjudication and clinical trial efficacy assessments compared with physician-reported end points. Although BICR is regularly used in clinical trials, it is often not feasible in clinical practice or research using RWD due to resource constraints; BICR is labor-intensive, time-consuming and costly. Where appropriate in observational studies, rwLugano may offer an alternative to outcome assessment without compromising construct validity.

## Summary points

- This study describes the development and validation of real-world Lugano (rwLugano), a real-world data (RWD)-based methodology for assessing lymphoma treatment response aligned with clinical trial standards.
- In collaboration with the US FDA, this retrospective, multisite chart abstraction study used data from the Cardinal Health Practice Research Network across US community oncology practices.
- The cohort included 178 adult patients with diffuse large B-cell lymphoma (DLBCL) initiating first-line therapy between 2015 and 2022.
- We compared three response assessment approaches – physician-charted responses from electronic health records (EHRs), rwLugano-derived responses per Lugano 2014 criteria and blinded independent central review (BICR).
- The rwLugano method showed higher agreement with BICR (87.9%, kappa [κ] = 0.52) than physician-charted responses (77.0%, κ = 0.40).
- Rates of complete response were 63.5% (physician-charted), 81.5% (rwLugano-derived) and 83.1% (BICR-adjudicated), indicating the closer alignment of rwLugano with trial-grade assessments.
- To ensure consistency, two radiologists independently reviewed the imaging scans and a medical oncologist adjudicated the discordant cases.
- Statistical analysis involved percentage concordance, κ, and multivariable generalized linear mixed modeling to evaluate agreement and predictors of complete response.
- As rwLugano enhances reproducibility and accuracy of real-world outcomes data, its use in clinical and regulatory decision-making is supported by this research.
- Adoption of rwLugano may reduce outcome misclassification and improve comparability between clinical trial data and real-world practice.

## Supplementary Material


