# Supplementary material for: A novel real-world data methodology for lymphoma outcome classification: the real-world Lugano study
Source: J Comp Eff Res. 2026 Mar 12;15(4):e250134. doi: 10.57264/cer-2025-0134 (PMC13044812; doi:10.57264/cer-2025-0134)
Supplement: Supplementary file 1 [file cer-15-250134-s1.pdf]

APPENDIX

Appendix Table 1: Lugano Criteria for Response Assessment (simplified)[1]

5-PS/Deauville Score	Change from Baseline	New Lesions	Bone Marrow	Treatment Response
1, 2, or 3	Reduced	No	No	CR
4 or 5	Reduced	No	Reduced	PR
4 or 5	No change	No	No change	NR/SD
4 or 5	Increased	No	Yes	PD
Any	Any	Yes	Any	PD

[1]Cheson BD, Fisher RI, Barrington SF, et al. Recommendations for initial evaluation, staging, and response assessment of Hodgkin and non-Hodgkin lymphoma: The Lugano classification. *Journal of Clinical Oncology* 2014;32(27):3059-3068.
Acronyms: 5-PS, 5-point scale; CR, complete response; NR/SD, no response/stable disease; PD, progressive disease; PR, partial response; SUV, standardized uptake value.
Legend: Based on the SUV of the most metabolically active lesion: 1) no uptake above background; 2) uptake ≤ mediastinum; 3) uptake > mediastinum but ≤ liver; 4) uptake moderately increased > liver; 5) markedly increased uptake above liver at any site and/or new lesions.

Appendix Table 2: Study Inclusion and Exclusion Criteria

Inclusion	Exclusion
1. ≥18 years of age at DLBCL diagnosis<br>2. Physician diagnosis of DLBCL, with histologic confirmation<br>3. Diagnosed between January 1, 2015, and December 31, 2022<br>4. Treated with an anthracycline-containing chemoimmunotherapy regimen that includes an anti-CD20 monoclonal antibody (ie, rituximab or obinutuzumab, with multiagent chemotherapy) as 1L therapy for DLBCL. Examples include but are not limited to:<br>a. R-CHOP<br>b. R-CHOEP<br>c. Dose-adjusted EPOCH-R<br>d. G-CHOP<br>5. PET/CT scans (≥2) available, including a baseline scan within 8 weeks prior to initiation of 1L therapy, and the initial 1L response assessment scan between 8 to 24 weeks after initiating 1L therapy<br>6. ≥6 months of follow-up from initiation of 1L therapy, though patients who were otherwise eligible but died within this period were included.	1. Patients undergoing treatment for other malignancies during 1L therapy<br>2. Patients with central nervous system metastasis<br>3. Patients enrolled in clinical trials during 1L treatment of DLBCL

Acronyms: 1L, first-line; CD20, cluster of differentiation 20; DLBCL, diffuse large B-cell lymphoma; EPOCH-R, etoposide, prednisone, vincristine, cyclophosphamide, doxorubicin, rituximab; G-CHOP, gemtuzumab ozogamicin, cyclophosphamide, doxorubicin, vincristine, prednisone; PET/CT, positron emission tomography/computed tomography; R-CHOEP, rituximab, cyclophosphamide, doxorubicin, vincristine, etoposide, prednisone; R-CHOP, rituximab, cyclophosphamide, doxorubicin, vincristine, prednisone.

Appendix Table 3: PRN Site Characteristics

**Practice type and size, n (%)**	
Small private community practice (up to 5 physicians)	2.0 (33.3)
Small private community practice (up to 5 physicians) owned by a hospital	0.0 (0.0)
Medium-sized private community practice (6-10 physicians)	0.0 (0.0)
Medium-sized private community practice (6-10 physicians) owned by a hospital	0.0 (0.0)
Large private community practice (>10 physicians)	4.0 (66.7)
**Number of study patients from each participating practice, n (%)**	
Practice 1	37.0 (20.8)
Practice 2	31.0 (17.4)
Practice 3	39.0 (21.9)
Practice 4	20.0 (11.2)
Practice 5	11.0 (6.2)
Practice 6	40.0 (22.5)
**Study site location, n (%)**	
South (AL, AR, DC, FL, GA, KY, LA, MS, NC, OK, SC, TN, TX, VA, WV)	3.0 (50.0)
Midwest (IA, IL, IN, KS, MI, MN, MO, ND, NE, OH, SD, WI)	1.0 (16.7)
Northeast (CT, DE, MA, MD, ME, NH, NJ, NY, PA, RI, VT)	1.0 (16.7)
West (AK, AZ, CA, CO, HI, ID, MT, NM, NV, OR, UT, WA, WY)	1.0 (16.7)
**Number of sites for each participating practice, n (%)**	
Practice 1	1.0 (16.7)
Practice 2	2.0 (33.3)
Practice 3	1.0 (16.7)
Practice 4	1.0 (16.7)
Practice 5	1.0 (16.7)
Practice 6	1.0 (16.7)
**Board-certified specialty of all physicians in participating sites, n (%)***	
Medical oncology	69.0 (48.3)
Hematology/Oncology	64.0 (44.8)
Gynecologic oncology	2.0 (1.4)
Pediatric hematology/oncology	0.0 (0.0)
Radiation oncology	4.0 (2.8)
Surgical oncology	2.0 (1.4)
Other (thoracic surgery and palliative care)	2.0 (1.4)

Acronyms: 1L, first-line; DLBCL, diffuse large B-cell lymphoma; max, maximum; min, minimum; P25-P75, 25th through 75th percentiles; PRN, Practice Research Network; SD, standard deviation.
Notes: *Not mutually exclusive.

Appendix Table 4: Comparing Agreement Between Deauville Score and rwDeauville and Between rwLugano-Derived With Deauville Versus rwDeauville Scores

**Agreement between physician-charted Deauville and rwDeauville scores among patients with Deauville scores at initial response scan (n = 148)**					
	**rwDeauville score**				
**Deauville score (among patients having scores)**	**1**	**2**	**3**	**4**	**5**
**1**	92	1	3	1	1
**2**	1	13	2	1	0
**3**	1	1	7	2	1
**4**	0	0	3	7	2
**5**	0	0	0	1	8
Overall percent agreement (total concordance/total sample)	87.6%				
Weighted kappa for 5-PS Deauville scores (κ, 95% CI)[1]	0.82 (0.74-0.90)				

**Agreement between rwLugano-derived scores using reported Deauville and rwDeauville scores at first response assessment (n = 148)**				
	**rwLugano-derived using rwDeauville score**			
**rwLugano-derived score using Deauville score**	**CR**	**PR**	**NR/SD**	**PD**
**CR**	121	4	0	2
**PR**	3	14	0	0
**NR/SD**	0	0	0	0
**PD**	0	0	0	4
Overall percent agreement (total concordance/total sample)	93.9%			
Weighted kappa for 5-PS Deauville scores (κ, 95% CI)[1]	0.77 (0.63 -0.91)			

Acronyms: 5-PS, 5-point scale; CR, complete response; κ, Cohen's kappa; NR/SD, no response/stable disease; PD, progressive disease; PR, partial response; rw, real-world.

Notes: [1]κ1 is the difference between rwLugano-derived and physician-charted k values and κ2 is the difference between rwLugano-derived and BICR-adjudicated k values.

Appendix Table 5: Univariate and Multivariable GLMM Analysis to Identify Predictors of Treatment Response Within 6 months from 1L Therapy Initiation: Agreement Between Physician-charted and rwLugano-derived, Physician-charted and BICR-adjudicated, and rwLugano-derived and BICR-adjudicated CR Assessments

	Physician-charted and rwLugano-derived CR[1]			Physician-charted and BICR-adjudicated CR[1]			rwLugano-derived and BICR-adjudicated CR[1]		
	OR	95% CI UL	95% CI LL	OR	95% CI UL	95% CI LL	OR	95% CI UL	95% CI LL
Age at 1L initiation, years (N= 178)	1.01	0.98	1.04	1.02	0.99	1.04	0.98	0.95	1.02
Time from DLBCL diagnosis to 1L initiation, weeks (N= 178)	0.98	0.95	1.02	1.07	0.91	1.25	0.94	0.79	1.12
**Insurance**									
Other (n= 71)	-	-	-	-	-	-	4.39	1.40	13.73
**Disease characteristics**									
Number of extranodal sites (N= 178)	3.60	1.59	8.12	1.92	1.09	3.36	-	-	-
**ECOG PS**									
Categoric (n=172)<br>2+/missing (n= 21)	0.26	0.08	0.84	-	-	-	-	-	-
**Stage at diagnosis**									
Categoric (n=154)									
Stage II (n= 42)	0.23	0.02	2.13	-	-	-	-	-	-
Stage III (n= 38)	0.09	0.01	0.81	-	-	-	-	-	-
Stage IV (n= 50)	0.05	0.07	0.48	-	-	-	-	-	-
Not available (n= 24)	0.07	0.01	0.69	-	-	-	-	-	-
***MYC* mutation**									
Unknown (n= 34)	-	-	-	0.41	0.17	0.98	0.74	0.24	2.30
Yes (n= 28)	-	-	-	0.38	0.15	0.98	0.26	0.09	0.76
**Comorbidities***									
Anemia (Yes [n=49])	-	-	-	0.37	0.17	0.82	-	-	-
Hypertension (Yes [n= 94])	2.80	1.20	6.53	-	-	-	-	-	-
Heart disease[2] (Yes [n= 38])	0.35	0.14	0.93	-	-	-	-	-	-

Acronyms: 1L, first-line; 5-PS, 5-point scale; BICR, blinded independent central review; CR, complete response; ECOG PS, Eastern Cooperative Oncology Group performance score; GLMM, generalized linear mixed model; NR/SD, no response/stable disease; OR, odds ratio; PD, progressive disease; PR, partial response; rw, real-world; SUV, standardized uptake value.

Treatment response end points:
Physician-charted response: 1L therapy response as charted in the medical record; the physician-charted response with the date closest to the scan date for rwLugano-derived and BICR-adjudicated assessments was used for pairwise comparison.
RwLugano-derived response: Calculated response based on Lugano classification components available on pretreatment (baseline) scans compared with scans on-treatment, with at least 1 on-treatment scan performed at initial response within 12 weeks; additional 1L on-treatment time points were evaluated as available for up to 6 months. The Lugano classification uses the terms complete metabolic response and partial metabolic response synonymously with CR and PR. In this study, CR and PR were used. The Lugano 5-PS is based on the SUV of the most metabolically active lesion: 1) no uptake above background; 2) uptake ≤ mediastinum; 3) uptake > mediastinum but ≤ liver; 4) uptake moderately increased > liver; 5) markedly increased uptake above liver at any site and/or new lesions.
BICR-adjudicated response: Assigned by two independent radiologists comparing pretreatment (baseline) scans with on-treatment scans, with at least 1 on-treatment (1L) scan performed at initial response within 12 weeks, using Lugano criteria; no additional clinical information was included in this assessment. Additional 1L on-treatment timepoints were evaluated as available for a given patient up to 6 months. Where there were discordant response classifications between the two independent radiologists, cases were reevaluated by both radiologists and discussed together to agree on 1 response classification for the on-treatment scan in question.
Notes: *Not mutually exclusive.
[1]We used GLMM analysis to determine the further impact of covariates and raters upon the level of agreement regarding predictors of treatment response. The model used a probit link for the binary categorical outcomes of agreement versus nonagreement for each scan. The initial model did not consider any additional covariates and treated both the patient and the raters as random effects. Subsequent models explored both additional random effects and conditional likelihood. Core random effects in the model were the patient as well the rater for each scan, with the site as a potential additional random effect. Conditional likelihood in the model included clinically and statistically significant variables based on the descriptive analysis and was expected to include age of the patient at study initiation, time between imaging scans, and tumor cell of origin. Additional clinical and demographic information was considered based on descriptive statistics in the primary and secondary analyses.
[2]Cardiovascular disease, congestive heart failure, coronary artery disease, or myocardial infarction.

Appendix Table 6: Univariate and Multivariable GLMM Analysis of Predictors of Concordance Between Physician-charted and rwLugano-derived Response With BICR-adjudicated Response Within 6 Months of 1L Therapy Initiation

**Agreement between physician-charted and rwLugano-derived CR (yes/no)[1]**	**Univariate OR (95% CI)**	**Multivariable OR (95% CI)**
**Reviewer (main independent variable)**		
BICR-adjudicated (N= 178)	Reference	Reference
Physician-charted (N= 178)	0.30 (0.17, 0.52)	0.23 (0.12, 0.43)
rwLugano-derived (N= 178)	1.16 (0.63, 2.15)	1.19 (0.61, 2.34)
Age at 1L initiation, years (N = 178)	1.01 (0.98, 1.03)	_
Time from DLBCL diagnosis to 1L initiation, weeks (N= 178)	0.99 (0.97, 1.01)	_
**Sex**		
Female (n= 73)	Reference	_
Male (n= 105)	1.44 (0.77, 2.68)	_
**Race and Ethnicity**		
Non-Hispanic White (n= 117)	Reference	_
Other (n = 61)	0.69 (0.36, 1.31)	_
**Insurance**		
Medicare/Medicaid (n= 107)	Reference	_
Other (n= 71)	1.26 (0.66, 2.37)	_
**Provider ID**		
02 (n= 37)	Reference	Reference
03 (n= 31)	2.45 (0.85, 7.07)	2.31 (0.63, 8.40)
04 (n= 39)	1.86 (0.72, 4.82)	2.31 (0.76, 7.04)
06 (n= 20)	2.00 (0.62, 6.51)	1.68 (0.44, 6.49)
07 (n= 11)	0.15 (0.04, 0.54)	0.12 (0.03, 0.52)
08 (n= 40)	1.06 (0.43, 2.62)	2.35 (0.77, 7.19)
**Study site location[2]**		
Midwest (n= 31)	Reference	_
West (n= 20)	0.81 (0.22, 3.02)	_
Northeast (n= 37)	0.40 (0.14, 1.19)	_
South (n= 90)	0.40 (0.16, 1.04)	_
**Disease characteristics**		
Number of extranodal sites (N= 178)	1.44 (0.97, 2.25)	_
Bulky disease	_	_
No (n= 143)	Reference	_

**Agreement between physician-charted and rwLugano-derived CR (yes/no)[1]**	**Univariate OR (95% CI)**	**Multivariable OR (95% CI)**
Yes (n= 35)	0.39 (0.19, 0.82)	_
Bone marrow involvement	_	_
No (n= 164)	Reference	_
Yes (n= 14)	1.19 (0.37, 3.81)	_
**ECOG PS score**		
**Categoric (n = 172)**		
0/1 (n= 157)	Reference	_
2+/missing (n= 21)	0.90 (0.35, 2.33)	_
Numeric (n= 172)	0.89 (0.56, 1.41)	_
**Stage at diagnosis (n = 154)**		
**Categoric**		
Stage I (n= 24)	Reference	Reference
Stage II (n= 42)	0.19 (0.05, 0.71)	0.16 (0.03, 0.71)
Stage III (n= 38)	0.43 (0.11, 1.68)	0.33 (0.07, 1.57)
Stage IV (n= 50)	0.15 (0.04, 0.54)	0.16 (0.04, 0.67)
Not available (n= 24)	0.11 (0.03, 0.42)	0.08 (0.02, 0.40)
Numeric (n= 154)	0.68 (0.49, 0.95)	_
**Cell of origin identified in report, n (%)**		
Germinal center B-cell like (n= 75)	Reference	_
None of the above (n= 24)	0.80 (0.31, 2.06)	_
Activated B-cell like (n= 11)	1.11 (0.29, 4.20)	_
Non-germinal center B-cell like (n= 55)	2.14 (0.98, 4.68)	_
Unclassified (n= 13)	0.66 (0.20, 2.15)	_
***BCL-2* mutation**		
No (n= 89)	Reference	_
Yes (n= 57)	0.66 (0.33, 1.32)	_
Unknown (n= 32)	0.67 (0.28, 1.57)	_
***BCL-6* mutation**		
No (n= 76)	Reference	_
Yes (n= 75)	0.97 (0.49, 1.91)	_
Unknown (n= 27)	0.73 (0.29, 1.82)	_
***MYC* mutation**		
No (n= 116)	Reference	_

Agreement between physician-charted and rwLugano-derived CR (yes/no)[1]	Univariate OR (95% CI)	Multivariable OR (95% CI)
Yes (n= 28)	0.68 (0.29, 1.60)	_
Unknown (n= 34)	0.64 (0.29, 1.41)	_
**Comorbidities***		
Anemia	_	_
No (n= 129)	Reference	Reference
Yes (n= 49)	0.42 (0.22, 0.82)	0.39 (0.17, 0.90)
Diabetes[3]	_	_
No (n= 142)	Reference	_
Yes (n= 36)	1.58 (0.71, 3.53)	_
Hypertension	_	_
No (n= 84)	Reference	_
Yes (n= 94)	1.58 (0.85, 2.94)	_
Dyslipidemia/Hypercholesterolemia	_	_
No (n= 124)	Reference	_
Yes (n= 54)	1.11 (0.57, 2.19)	_
Heart disease[4]		
No (n= 140)	Reference	Reference
Yes (n= 38)	0.38 (0.18, 0.76)	0.25 (0.11, 0.59)
Mental illness[5]	_	_
No (n= 152)	Reference	_
Yes (n= 26)	0.54 (0.23, 1.24)	_
Joint disorders[6]	_	_
No (n= 139)	Reference	_
Yes (n= 39)	0.98 (0.46, 2.06)	_

Acronyms: 1L, first-line; 5-PS, 5-point scale; BICR, blinded independent central review; CR, complete response; ECOG PS, Eastern Cooperative Oncology Group performance score; GLMM, generalized linear mixed model; NR/SD, no response/stable disease; OR, odds ratio; P25-P75, 25th through 75th percentiles; PD, progressive disease; PR, partial response; rw, real-world; SUV, standardized uptake value.

Treatment response end points:

Physician-charted response: 1L therapy response as charted in the medical record; the physician-charted response with the date closest to the scan date for rwLugano-derived and BICR-adjudicated assessments was used for pairwise comparison.

RwLugano-derived response: Calculated response based on Lugano classification components available on pretreatment (baseline) scans compared with scans on-treatment, with at least 1 on-treatment scan performed at initial response within 12 weeks; additional 1L on-treatment time points were evaluated as available for up to 6 months. The Lugano classification uses the terms complete

metabolic response and partial metabolic response synonymously with CR and PR. In this study, CR and PR were used. The Lugano 5-PS is based on the SUV of the most metabolically active lesion: 1) no uptake above background; 2) uptake ≤ mediastinum; 3) uptake > mediastinum but ≤ liver; 4) uptake moderately increased > liver; 5) markedly increased uptake above liver at any site and/or new lesions.

BICR-adjudicated response: Assigned by two independent radiologists comparing pretreatment (baseline) scans with on-treatment scans, with at least 1 on-treatment (1L) scan performed at initial response within 12 weeks, using Lugano criteria; no additional clinical information was included in this assessment. Additional 1L on-treatment timepoints were evaluated as available for a given patient up to 6 months. Where there were discordant response classifications between the two independent radiologists, cases were reevaluated by both radiologists and discussed together to agree on 1 response classification for the on-treatment scan in question.

Notes: *Not mutually exclusive.

[1]We used GLMM analysis to determine the further impact of covariates and raters upon the level of agreement regarding predictors of treatment response. The model used a probit link for the binary categorical outcomes of agreement versus nonagreement for each scan. The initial model did not consider any additional covariates and treated both the patient and the raters as random effects. Subsequent models explored both additional random effects and conditional likelihood. Core random effects in the model were the patient as well the rater for each scan, with the site as a potential additional random effect. Conditional likelihood in the model included clinically and statistically significant variables based on the descriptive analysis and was expected to include age of the patient at study initiation, time between imaging scans, and tumor cell of origin. Additional clinical and demographic information was considered based on descriptive statistics in the primary and secondary analyses.

[2]Northeast includes Connecticut, Delaware, Massachusetts, Maine, Maryland, New Hampshire, New Jersey, New York, Pennsylvania, Rhode Island, Vermont; Midwest includes Iowa, Illinois, Indiana, Kansas, Michigan, Minnesota, Missouri, North Dakota, Nebraska, Ohio, South Dakota, Wisconsin; South includes Alabama, Arkansas, District of Columbia, Florida, Georgia, Kentucky, Louisiana, Mississippi, North Carolina, Oklahoma, South Carolina, Tennessee, Texas, Virginia, West Virginia; West includes Alaska, Arizona, California, Colorado, Hawaii, Idaho, Montana, New Mexico, Nevada, Oregon, Utah, Washington, Wyoming.

[3]With and without chronic complications.

[4]Cardiovascular disease, congestive heart failure, coronary artery disease, or myocardial infarction.

[5]Anxiety, depression, or dementia.

[6]Arthritis or gout.

Appendix Table 7: Subgroup Analyses of Agreement Between Physician-charted and BICR-adjudicated Response Based on BICR Concordance

	BICR-adjudicated response			
**Physician-charted response**	**CR**	**PR**	**NR/SD**	**PD**
**CR**	91	3	0	0
**PR**	30	15	1	1
**NR/SD**	1	1	1	1
**PD**	0	1	0	1

**Agreement (κ, 95% CI, testing κ = 0, 95% CI)**	κ	**95% CI Lower Limit**	**95% CI Upper Limit**	**95% CI Lower Limit for Testing κ = 0**	**95% CI Upper Limit for Testing κ = 0**
CR	0.43	0.29	0.58	0.29	0.58
PR	0.32	0.16	0.47	0.18	0.46
NR/SD	0.32	-0.17	0.81	0.17	0.47
PD	0.39	-0.16	0.94	0.23	0.55
ORR[1]	0.53	0.16	0.89	0.37	0.69
**GLMM OR (CR) physician-charted vs BICR-adjudicated**					
Weighted κ, 95% CI for 4-level response[2]	0.44	0.30	0.58	0.32	0.56
Overall percent agreement (total concordance/total sample)	73.5%				

Acronyms: BICR, blinded independent central review; CR, complete response; GLMM, generalized linear mixed model; NR/SD, no response/stable disease; OR, odds ratio; ORR, objective response rate; PD, progressive disease; PR, partial response.

Notes:
[1]ORR calculated using sum of patients with CR or PR divided by total patients.
[2]κ1 is the difference between rwLugano-derived and physician-charted values and κ2 is the difference between rwLugano-derived and BICR-adjudicated values.

Appendix Table 8: Subgroup Analyses of Agreement Between BICR-adjudicated and rwLugano-derived Response Based on BICR Concordance

	rwLugano-derived response			
**BICR-adjudicated response**	**CR**	**PR**	**NR/SD**	**PD**
**CR**	114	7	0	1
**PR**	7	10	1	2
**NR/SD**	2	0	0	0
**PD**	1	0	0	2

**Agreement (κ, 95% CI, testing κ = 0, 95% CI)**	**κ**	**95% CI-Lower Limit**	**95% CI-Upper Limit**	**95% CI-Lower Limit for Testing κ = 0**	**95% CI-Upper Limit for Testing κ = 0**
CR	0.55	0.37	0.74	0.39	0.71
PR	0.47	0.26	0.69	0.31	0.64
NR/SD	-0.01	-0.02	0.00	-0.16	0.14
PD	0.49	0.06	0.92	0.33	0.64
ORR[1]	0.34	-0.03	0.71	0.18	0.50
**GLMM OR (CR) rwLugano-derived vs BICR-adjudicated**					
Weighted κ, 95% CI for 4-level response[2]	0.50	0.31	0.70	0.38	0.63
Overall percent agreement (total concordance/total sample)	85.7%				

Acronyms: BICR, blinded independent central review; CR, complete response; GLMM, generalized linear mixed model; NR/SD, no response/stable disease; OR, odds ratio; ORR, objective response rate; PD, progressive disease; PR, partial response.

Notes:
[1]ORR calculated using sum of patients with CR or PR divided by total patients.
[2]κ1 is the difference between rwLugano-derived and physician-charted values and κ2 is the difference between rwLugano-derived and BICR-adjudicated values.

Appendix Table 9: Subgroup Analyses of Agreement Between Physician-charted and rwLugano-derived Response Based on BICR Concordance

	rwLugano-derived response			
**Physician-charted response**	**CR**	**PR**	**NR/SD**	**PD**
**CR**	94	0	0	0
**PR**	28	17	0	2
**NR/SD**	2	0	1	1
**PD**	0	0	0	2

**Agreement (κ, 95% CI, testing κ = 0 , 95% CI)**	κ	**95% CI-Lower Limit**	**95% CI-Upper Limit**	**95% CI-Lower Limit for Testing κ = 0**	**95% CI-Upper Limit for Testing κ = 0**
CR	0.50	0.36	0.63	0.36	0.63
PR	0.44	0.29	0.58	0.30	0.57
NR/SD	0.39	-0.15	0.93	0.26	0.52
PD	0.56	0.12	1.00	0.42	0.71
ORR[1]	0.65	0.33	0.97	0.49	0.81
**GLMM OR (CR) physician-charted vs rwLugano-derived**					
Weighted κ, 95% CI for 4-level response[2]	0.52	0.38	0.67	0.41	0.64
Overall percent agreement (total concordance/total sample)	77.5%				

Acronyms: BICR, blinded independent central review; CR, complete response; GLMM, generalized linear mixed model; NR/SD, no response/stable disease; OR, odds ratio; ORR, objective response rate; PD, progressive disease; PR, partial response.

Notes:

[1]ORR calculated using sum of patients with CR or PR divided by total patients.

[2]κ1 is the difference between rwLugano-derived and physician-charted values and κ2 is the difference between rwLugano-derived and BICR-adjudicated values.

Appendix Figure 1: rwDeauville Methodology

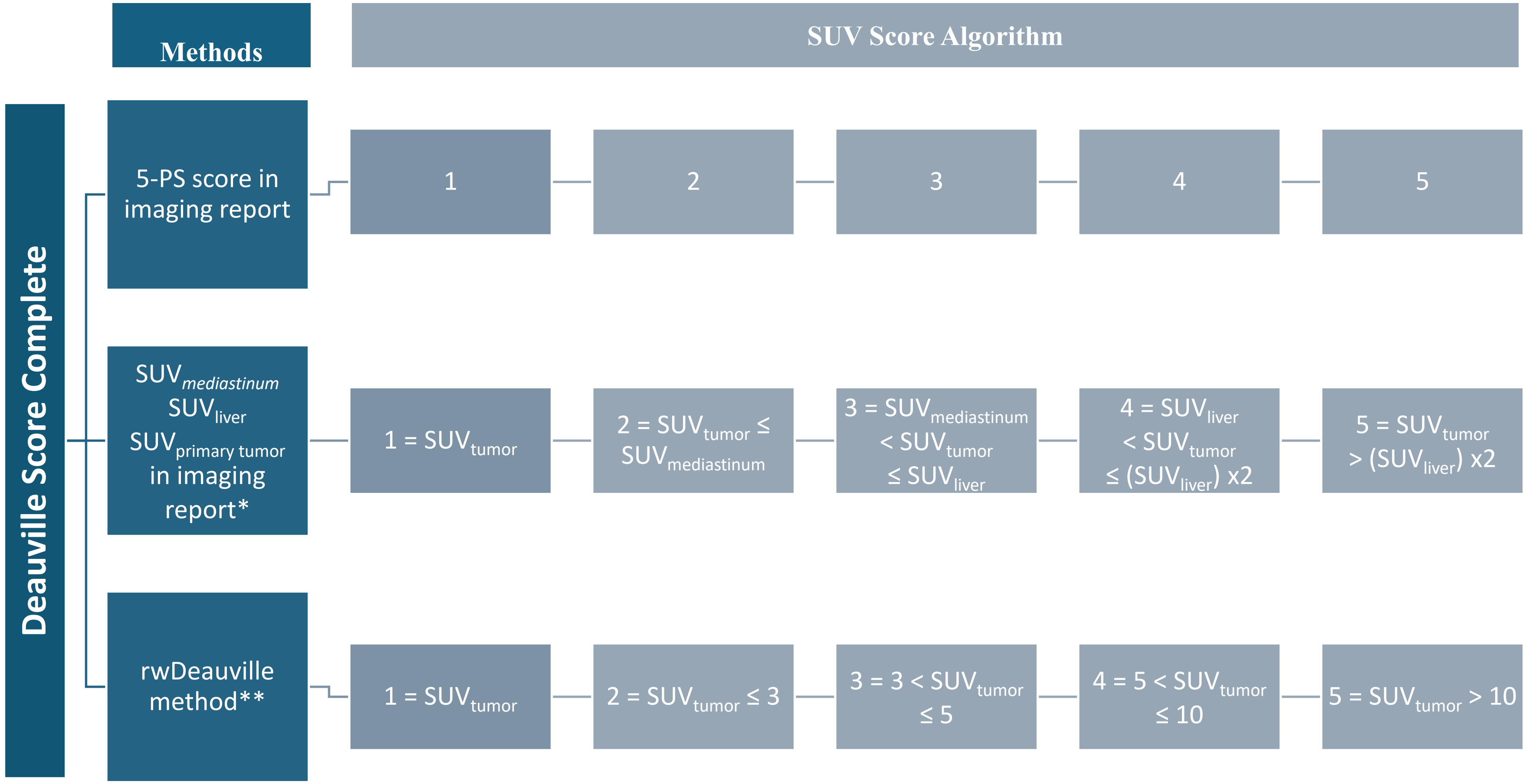

Figure Legend

5-PS/ Deauville	Description	SUV Algorithm for Deauville*	SUV Algorithm for rwDeauville**
1	No uptake or no residual uptake (when used interim)	$SUV_{tumor} = 1$	$SUV_{tumor} = 1$
2	Slight uptake, but ≤ blood pool (mediastinum)	$SUV_{tumor} \leq SUV_{mediastinum}$	$SUV_{tumor} \leq 3$
3	Uptake > mediastinal, but ≤ uptake in the liver	$SUV_{mediastinum} < SUV_{tumor} \leq SUV_{liver}$	$3 < SUV_{tumor} \leq 5$
4	Uptake slightly to moderately higher than liver	$SUV_{liver} < SUV_{tumor} \leq (SUV_{liver})$ x2	$5 < SUV_{tumor} \leq 10$
5	Markedly increased uptake or any new lesion (on response evaluation)	$SUV_{tumor} > (SUV_{liver})$ x2	$SUV_{tumor} > 10$

Acronyms: 5-PS, 5-point scale; rw, real-world; SUV, standardized uptake value.

Notes:
*Calculated when SUV data for background, mediastinum, liver, and tumor are complete.
**When SUV of background, mediastinum, or liver is missing, SUV=1 for background, SUV=3 for mediastinum, and SUV=5 for liver.
